# The application of augmented reality in robotic general surgery: A mini-review

**DOI:** 10.1515/med-2025-1170

**Published:** 2025-04-01

**Authors:** Gian Luigi Canu, Fabio Medas, Eleonora Noli, Giacomo Calini, Matteo Rottoli, Alessandra Ruggeri, Federico Cappellacci, Pietro Giorgio Calò

**Affiliations:** Department of Surgical Sciences, University of Cagliari, Monserrato, CA, Italy; Surgery of the Alimentary Tract, IRCCS Azienda Ospedaliero-Universitaria di Bologna, Bologna, Italy; Department of Medical and Surgical Sciences, Alma Mater Studiorum University of Bologna, Bologna, Italy; Cellular Signalling Laboratory, Anatomy Center, Department of Biomedical and Neuromotor Sciences (DIBINEM), University of Bologna, Bologna, Italy

**Keywords:** general surgery, robotic surgery, human–robot interaction, imaging technologies, augmented reality

## Abstract

In robotic surgery, surgical planning and surgical navigation represent two crucial elements, allowing surgeons to maximize surgical outcomes while minimizing the risk of complications. In this context, an emerging imaging technology, namely augmented reality (AR), can represent a powerful tool to create an integration of preoperative 3D models into the live intraoperative view, providing an interactive visual interface rather than a simple operative field. In this way, surgeons can be guided by preoperative imaging during the operation. This makes the surgical procedure more accurate and safer, leading to so-called “precision surgery”. This article aims to provide an overview of developments in the application of AR in robotic general surgery. The integration of this imaging technology in this surgical field is showing promising results. The main benefits include improved oncological outcomes and reduced occurrence of complications. In addition, its application may also be important for surgical education. However, we are still in the initial phase of the experience and some important limitations remain. Moreover, to our knowledge, to date, reports in the literature regarding the integration of AR in robotic general surgery are still very limited. To improve its application, close collaboration between engineers, software developers, and surgeons is mandatory.

## 1 Introduction

In the last few decades, in the field of general surgery, minimally invasive approaches have been increasingly used.

In this context, laparoscopic surgery has amply demonstrated its advantages, such as reduced postoperative pain and shorter recovery, with comparable results compared with open surgery in terms of complications and oncological outcomes. However, laparoscopy, especially regarding challenging surgical procedures, has some disadvantages, such as limited maneuverability. Laparoscopic instruments are rigid and can be opened and closed to grasp or cut, allowing five degrees of freedom. In this field, improvements in instrumentation have lagged behind clinical developments [1,2,3,4,5,6].

More recently, robotic surgery has been able to overcome some of the limitations of the laparoscopic approach [7,8,9,10,11,12,13]. The robotic platforms allow the combination of the multiple advantages of laparoscopy and the dexterity of the open approach, also offering better visualization and ergonomics. Concerning robotic instruments, they are equipped with a miniaturized wrist that allows them to achieve seven degrees of freedom [14,15].

In consideration of its advantages, the use of the robot in surgery is gradually extending to almost all surgical specialties [16,17,18,19]. The main obstacle to the wide diffusion of robotic surgery is represented by the high costs, both direct (related to the robotic platforms and necessary instrumentation) and indirect (related to operative times, which are longer) [20].

In robotic surgery, surgical planning and surgical navigation represent two crucial elements, allowing surgeons to maximize surgical outcomes while minimizing the risk of complications. In this context, an emerging imaging technology, namely augmented reality (AR), can represent a powerful tool to create, by means of dedicated software, which can be supported by robotic platforms, an integration of preoperative 3D models into the live intraoperative view, providing an interactive visual interface rather than a simple operative field. In this way, surgeons can be guided by preoperative imaging during the operation [21,22,23,24,25,26,27]. This makes the surgical procedure more accurate and safer, leading to so-called “precision surgery”. The emerging concept of “precision medicine”, in this case “precision surgery”, aims to personalize treatment for each individual patient with each specific disease [28,29].

This article aims to provide an overview of developments in the application of AR in robotic general surgery.

## 2 General principles on AR

The most common definition of AR, enunciated by Azuma in 1997, states that in AR “3D virtual objects are integrated into a 3D real environment in real-time” [30]. In fact, this imaging technology allows to improve the perception of the real world by superimposing virtual images on it.

It is important to distinguish AR from two other similar but different imaging technologies: virtual reality (VR) and mixed reality (MR). VR transports users to a completely different and immersive environment. Unlike AR (and even MR), this imaging technology isolates users from the real world, providing an entirely artificial experience. MR makes a step forward from AR by combining the virtual and physical worlds in a way that enables virtual objects to interact with the real environment. Through this imaging technology, users not only see the digital content but also can manipulate and interact with it as if it is part of their surroundings [23].

An AR environment can be experienced by means of various technologies, such as computer monitors, mobile displays (smartphone or tablet screens), head-mounted displays, and the recently introduced AR goggles (e.g., Google Glass and Microsoft Hololens) [23].

From a historical point of view, AR dates back to the invention of VR in the 1960s. In those years, Sutherland introduced the concept of “Ultimate Display”, which stands for the simulation of a synthetic environment similar to the actual reality [23,31]. Subsequently, Milgram and Kishino introduced the “virtuality continuum” concept, which created the connection between the real world and a completely virtual one [23,32].

Over the last decades, AR has been used in various areas to improve visual feedback from information systems, becoming a popular multidisciplinary research topic. The continuous developments in the field of technology (represented, for example, by advanced cameras, faster computers, and novel algorithms) increasingly motivate researchers to broaden the application areas of AR, overcoming its limitations. Sensing errors and registration issues are considered the most important challenges of AR technology [23,33].

In recent years, several applications of AR have been developed in the field of robotics. In this context, AR can act as a new tool for information exchange with autonomous systems, enhancing the effectiveness of the interaction between humans and robots. Robots are progressively becoming omnipresent in everyday life, expanding their conventional use in industry to other areas, including the field of medicine [23].

## 3 AR and robotic surgery

In medicine, and in particular in the field of surgery, the application of AR existed before the introduction of robotic surgery.

In the early 2000s, AR was applied in laparoscopic operations, in various surgical branches. In this context, collaboration between engineers, software developers, and surgeons has led to progressive developments in this field of minimally invasive surgery [34,35,36,37,38,39].

However, robotic surgery represents the area in which AR may have the best application. In fact, although this imaging technology may also be used during laparoscopy, in robotic surgery surgeons are able to interact directly with the computer (robotic platform), greatly facilitating the integration [23,24,40].

### 3.1 Usefulness during the surgical procedure

During robotic surgery, as mentioned above, AR can provide valuable help, even to already experienced surgeons. In fact, when the preoperative 3D reconstruction of the surgical target is accurately superimposed on the surgical field, the interpretation of intraoperative anatomy is considerably facilitated. This simplifies decision-making processes during the operation and improves operative precision, allowing safer surgery [21,22,23,41,42,43].

In the literature, there are some studies reporting the application, in different surgical specialties, of AR in robotic surgery. Liu et al. [44] described this integration in transoral robotic surgery to perform base-of-tongue tumor resection. In craniomaxillofacial surgery, this application was documented by Lin et al. [45]. Wang et al. [46] reported an AR navigation system in oral and maxillofacial surgery. In robotic mandibular plastic surgery, the integration of AR was described by Zhou et al. [47]. Roberts et al. [26] and Porpiglia et al. [48] documented the use of AR technology during urological robot-assisted procedures (such as prostatectomy and partial nephrectomy). In the field of neurosurgery, various applications of AR in robotic systems are reported [49].

In relation to the different surgical specialties, it is important to emphasize that the accuracy of virtual image superposition is completely different if this imaging technology is applied in surgical procedures involving rigid and immobile structures or in operations involving mobile and deformable structures [36,50].

### 3.2 Usefulness in surgical education

The integration of AR into robotic platforms can also potentially be very useful for surgical education [50,51].

This imaging technology may represent a useful tool for residents or less-experienced surgeons to better identify anatomical structures, thus making it easier to perform operations. Moreover, for the same reason, AR can be used to train surgeons in new procedures [50,51].

It is important to emphasize that through the application of AR in robotic surgery, instead of passive observation of the operations, real-time trainee teaching is possible [21,50,51].

In laparoscopic surgery, AR-based surgical training appears to accelerate the acquisition of simple skills (such as suturing) and even reduce the learning curve in more complex operations [21,52,53].

In robotic surgery, similar applications in surgical education are lacking, with only a few experiences reported [21,54,55].

### 3.3 Limitations

Although the advantages related to the application of AR in robotic surgery are clear, its use in clinical practice is still very limited [21,22,56,57,58].

The main limitation hindering the wide extension of this integration, particularly in some surgical specialties, is represented by the continuous misalignment of 3D models with respect to the real surgical field. Factors causing the misalignment include the interference due to surgical instruments, the mobility and deformability of some organs, and the interference caused by breathing movements [21,22,58].

Current research in this field is focused on the development, by employing more efficient deep learning algorithms, of an automated overlay system that allows the automatic anchoring of virtual models to the real organs. However, significant improvements have not yet been achieved [21,22,40,58,59,60].

## 4 AR in robotic general surgery

The application of AR technology can provide valuable help in the field of general surgery. For example, in a cholecystectomy, where bile duct injury, associated with anatomical distortion due to aberrant anatomy or inflammation, may be a disastrous complication, this imaging technology, with an intraoperative overlay of safe dissection areas, can be a very useful tool for improving surgical outcomes [61].

However, the application of AR in robotic general surgery is still very limited compared to other surgical fields, such as urology, otolaryngology, neurosurgery, maxillofacial surgery, and orthopedics [21,22,23,62,63,64]. This difference is mainly due to the mobility and deformability of the target organs, as described above.

The branch of general surgery in which the application of this imaging technology is developing most is robotic liver surgery. In this context, AR may allow to overcome the widely described limitations in performing liver resections with this approach. In fact, despite the enhanced video resolution and the sense of depth obtained through the 3D visualization, robotic liver surgery has some of the disadvantages of laparoscopic surgery compared to the open approach [22,23,65,66].

The main issue in this context is certainly represented by the absence of tactile feedback, which helps the surgeon to orient himself during parenchymal transection. In this regard, the integration of AR into robotic platforms can be useful for the accurate and safe identification of vascular structures, thus helping in the mapping of resection plans [22,67].

Moreover, from an oncological point of view, the application of AR technology helps to localize the tumor [22,68]. An intraoperative CT or ultrasound guidance can also be utilized, however, with the drawback of radiation, the constant shifting of instruments, or the occasional difficulty in locating lesions [22].

Another benefit of the application of AR in robotic liver surgery is port positioning, which represents a crucial step of the operation. The projection of a virtual image of the liver on the surface of the skin before beginning the surgical procedure enables accurate targeting of the liver structures and the lesion, allowing optimal port placement. This makes surgical maneuvers easier [22,67].

From a general point of view, regarding the indications for the application of AR in robotic surgery, as this integration is at an early stage of development, they are not yet well defined. However, we believe that once the limitations are overcome, the indications for its application will expand more and more. In fact, the application of this imaging technology in robotic general surgery has theoretically unlimited potential and can lead to a considerable simplification of surgical procedures.

## 5 Conclusions

In the era of “precision medicine”, robotic surgery represents an excellent field for the application of AR in clinical practice.

The integration of this imaging technology in robotic general surgery is showing promising results. The main benefits include improved oncological outcomes and reduced occurrence of complications. In addition, its application may also be important for surgical education.

However, we are still at the initial phase of the experience and some important limitations remain, due to the limited spread of robotic platforms and the still insufficient technological development. Moreover, to our knowledge, to date, reports in the literature regarding the integration of this imaging technology in robotic general surgery are still very limited. To improve its application, close collaboration between engineers, software developers, and surgeons is mandatory.

## References

[1] Kasai M, Cipriani F, Gayet B, Aldrighetti L, Ratti F, Sarmiento JM, et al. Laparoscopic versus open major hepatectomy: A systematic review and meta-analysis of individual patient data. Surgery. 2018;163(5):985–95. 10.1016/j.surg.2018.01.020. 29555197

[2] Fiorentini G, Zironda A, Calini G, Abdalla S, Nagorney DM, Warner SG, et al. Minimally invasive vs open approach to the simultaneous treatment of colorectal tumors with synchronous liver metastasis: A single center, propensity-score matched analysis from Mayo clinic. HPB (Oxf). 2023;25(11):1337–44. 10.1016/j.hpb.2023.06.010. 37626006

[3] Guerrini GP, Esposito G, Tarantino G, Serra V, Olivieri T, Catellani B, et al. Laparoscopic versus open liver resection for intrahepatic cholangiocarcinoma: the first meta-analysis. Langenbecks Arch Surg. 2020;405(3):265–75. 10.1007/s00423-020-01877-0. 32367395

[4] Calini G, Abdalla S, Aziz MAAE, Behm KT, Shawki SF, Mathis KL, et al. Incisional hernia rates between intracorporeal and extracorporeal anastomosis in minimally invasive ileocolic resection for Crohn’s disease. Langenbecks Arch Surg. 2023;408(1):251. 10.1007/s00423-023-02976-4. 37382678

[5] Syn NL, Kabir T, Koh YX, Tan HL, Wang LZ, Chin BZ, et al. Survival advantage of laparoscopic versus open resection for colorectal liver metastases: A meta-analysis of individual patient data from randomized trials and propensity-score matched studies. Ann Surg. 2020;272(2):253–65. 10.1097/SLA.0000000000003672. 32675538

[6] Schmelzle M, Krenzien F, Schöning W, Pratschke J. Laparoscopic liver resection: indications, limitations, and economic aspects. Langenbecks Arch Surg. 2020;405(6):725–35. 10.1007/s00423-020-01918-8. PMC7471173 32607841

[7] Calini G, Abdalla S, Abd El Aziz MA, Merchea A, Larson DW, Behm KT. Ileocolic resection for Crohn’s disease: Robotic intracorporeal compared to laparoscopic extracorporeal anastomosis. J Robot Surg. 2023;17(5):2157–66. 10.1007/s11701-023-01635-6. 37264221

[8] Gavriilidis P, Roberts KJ, Aldrighetti L, Sutcliffe RP. A comparison between robotic, laparoscopic and open hepatectomy: A systematic review and network meta-analysis. Eur J Surg Oncol. 2020;46(7):1214–24. 10.1016/j.ejso.2020.03.227. 32312592

[9] Zhao Z, Yin Z, Li M, Jiang N, Liu R. State of the art in robotic liver surgery: A meta-analysis. Updates Surg. 2021;73(3):977–87. 10.1007/s13304-020-00906-3. 33146887

[10] Zhang L, Yuan Q, Xu Y, Wang W. Comparative clinical outcomes of robot-assisted liver resection versus laparoscopic liver resection: A meta-analysis. PLoS One. 2020;15(10):e0240593. 10.1371/journal.pone.0240593. PMC7553328 33048989

[11] Rottoli M, Violante T, Calini G, Cardelli S, Novelli M, Poggioli G. A multi-docking strategy for robotic LAR and deep pelvic surgery with the Hugo RAS system: experience from a tertiary referral center. Int J Colorectal Dis. 2024;39(1):154. 10.1007/s00384-024-04728-2. PMC11442597 39349880

[12] Tsilimigras DI, Moris D, Vagios S, Merath K, Pawlik TM. Safety and oncologic outcomes of robotic liver resections: A systematic review. J Surg Oncol. 2018;117(7):1517–30. 10.1002/jso.25018. 29473968

[13] Liu R, Wakabayashi G, Kim HJ, Choi GH, Yiengpruksawan A, Fong Y, et al. International consensus statement on robotic hepatectomy surgery in 2018. World J Gastroenterol. 2019;25(12):1432–44. 10.3748/wjg.v25.i12.1432. PMC6441912 30948907

[14] Marohn MR, Hanly EJ. Twenty-first century surgery using twenty-first century technology: surgical robotics. Curr Surg. 2004;61(5):466–73. 10.1016/j.cursur.2004.03.009. 15475097

[15] Muaddi H, Hafid ME, Choi WJ, Lillie E, de Mestral C, Nathens A, et al. Clinical outcomes of robotic surgery compared to conventional surgical approaches (Laparoscopic or Open): A systematic overview of reviews. Ann Surg. 2021;273(3):467–73. 10.1097/SLA.0000000000003915. 32398482

[16] Fregoli L, Rossi L, Papini P, Materazzi G. Robotic transaxillary thyroidectomy: State of the art. Gland Surg. 2020;9(Suppl 1):S61–4. 10.21037/gs.2019.10.11. PMC6995899 32055500

[17] Materazzi G, Rossi L. Robot-assisted adrenalectomy: State of the art. Updates Surg. 2021;73(3):1131–46. 10.1007/s13304-020-00915-2. PMC8184704 33175318

[18] Pennestrì F, Sessa L, Prioli F, Gallucci P, Ciccoritti L, Greco F, et al. Robotic vs laparoscopic approach for single anastomosis duodenal-ileal bypass with sleeve gastrectomy: A propensity score matching analysis. Updates Surg. 2023;75(1):175–87. 10.1007/s13304-022-01381-8. PMC9834101 36161395

[19] De Crea C, Pennestrì F, Voloudakis N, Sessa L, Procopio PF, Gallucci P, et al. Robot-assisted vs laparoscopic lateral transabdominal adrenalectomy: A propensity score matching analysis. Surg Endosc. 2022;36(11):8619–29. 10.1007/s00464-022-09663-3. PMC9613740 36190555

[20] Mazzone E, Mistretta FA, Knipper S, Tian Z, Larcher A, Widmer H, et al. Contemporary national assessment of robot-assisted surgery rates and total hospital charges for major surgical uro-oncological procedures in the United States. J Endourol. 2019;33(6):438–47. 10.1089/end.2018.0840. 30931607

[21] Franco A, Amparore D, Porpiglia F, Autorino R. Augmented reality-guided robotic surgery: Drilling down a giant leap into small steps. Eur Urol. 2023;84(1):92–4. 10.1016/j.eururo.2023.03.021. 37032185

[22] Giannone F, Felli E, Cherkaoui Z, Mascagni P, Pessaux P. Augmented reality and image-guided robotic liver surgery. Cancers (Basel). 2021;13(24):6268. 10.3390/cancers13246268. PMC8699460 34944887

[23] Makhataeva Z, Varol HA. Augmented reality for robotics: A review. Robotics. 2020;9(2):21. 10.3390/robotics9020021.

[24] Zhang C, Hallbeck MS, Salehinejad H, Thiels C. The integration of artificial intelligence in robotic surgery: A narrative review. Surgery. 2024;176(3):552–7. 10.1016/j.surg.2024.02.005. 38480053

[25] Andras I, Mazzone E, van Leeuwen FWB, De Naeyer G, van Oosterom MN, Beato S, et al. Artificial intelligence and robotics: A combination that is changing the operating room. World J Urol. 2020;38(10):2359–66. 10.1007/s00345-019-03037-6. 31776737

[26] Roberts S, Desai A, Checcucci E, Puliatti S, Taratkin M, Kowalewski KF, et al. Augmented reality” applications in urology: A systematic review. Minerva Urol Nephrol. 2022;74(5):528–37. 10.23736/S2724-6051.22.04726-7. 35383432

[27] Giménez M, Gallix B, Costamagna G, Vauthey JN, Moche M, Wakabayashi G, et al. Definitions of computer-assisted surgery and intervention, image-guided surgery and intervention, hybrid operating room, and guidance systems: Strasbourg International Consensus Study. Ann Surg Open. 2020;1(2):e021. 10.1097/AS9.0000000000000021. PMC7771637 33392607

[28] Autorino R, Porpiglia F, Dasgupta P, Rassweiler J, Catto JW, Hampton LJ, et al. Precision surgery and genitourinary cancers. Eur J Surg Oncol. 2017;43(5):893–908. 10.1016/j.ejso.2017.02.005. 28254473

[29] Veronesi U, Stafyla V, Luini A, Veronesi P. Breast cancer: From “maximum tolerable” to “minimum effective” treatment. Front Oncol. 2012;2:125. 10.3389/fonc.2012.00125. PMC3465814 23061042

[30] Azuma RT. A survey of augmented reality. Presence: Teleoperators Virtual Environ. 1997;6(4):355–85. 10.1162/pres.1997.6.4.355.

[31] Sutherland IE. The ultimate display. Proceedings of the IFIP Congress. London, UK: Macmillan and Co.; 1965. p. 506–8.

[32] Milgram P, Kishino F. A taxonomy of mixed reality visual displays. IEICE Trans Inf Syst. 1994;77:1321–9.

[33] Azuma R, Baillot Y, Behringer R, Feiner S, Julier S, MacIntyre B. Recent advances in augmented reality. IEEE Comput Graph Appl. 2001;21(6):34–47. 10.1109/38.963459.

[34] Marescaux J, Rubino F, Arenas M, Mutter D, Soler L. Augmented-reality-assisted laparoscopic adrenalectomy. JAMA. 2004;292(18):2214–5. 10.1001/jama.292.18.2214-c. 15536106

[35] Konishi K, Hashizume M, Nakamoto M, Kakeji Y, Yoshino I, Taketomi A, et al. Augmented reality navigation system for endoscopic surgery based on three-dimensional ultrasound and computed tomography: application to 20 clinical cases. Int Congr Ser. 2005;1281:537–42. 10.1016/j.ics.2005.03.234.

[36] Docea R, Xu J, Ling W, Jenke AC, Kolbinger FR, Distler M, et al. SeeSaw: Learning soft tissue deformation from laparoscopy videos with GNNs. IEEE Trans Biomed Eng. 2024;71(12):3432–45. 10.1109/TBME.2024.3424771. 39008390

[37] Ribeiro M, Espinel Y, Rabbani N, Pereira B, Bartoli A, Buc E. Augmented reality guided laparoscopic liver resection: A phantom study with intraparenchymal tumors. J Surg Res. 2024;296:612–20. 10.1016/j.jss.2023.12.014. 38354617

[38] Ali S, Espinel Y, Jin Y, Liu P, Güttner B, Zhang X, et al. An objective comparison of methods for augmented reality in laparoscopic liver resection by preoperative-to-intraoperative image fusion from the MICCAI2022 challenge. Med Image Anal. 2025;99:103371. 10.1016/j.media.2024.103371. 39488186

[39] Ramalhinho J, Yoo S, Dowrick T, Koo B, Somasundaram M, Gurusamy K, et al. The value of augmented reality in surgery – A usability study on laparoscopic liver surgery. Med Image Anal. 2023;90:102943. 10.1016/j.media.2023.102943. PMC10958137 37703675

[40] Kolbinger FR, Bodenstedt S, Carstens M, Leger S, Krell S, Rinner FM, et al. Artificial Intelligence for context-aware surgical guidance in complex robot-assisted oncological procedures: An exploratory feasibility study. Eur J Surg Oncol. 2024;50(12):106996. 10.1016/j.ejso.2023.106996. 37591704

[41] Diana M, Marescaux J. Robotic surgery. Br J Surg. 2015;102(2):e15–28. 10.1002/bjs.9711. 25627128

[42] Navab N, Fellow M, Hennersperger C, Frisch B, Fürst B. Personalized, relevance-based multimodal robotic imaging and augmented reality for computer assisted interventions. Med Image Anal. 2016;33:64–71. 10.1016/j.media.2016.06.021. 27475417

[43] Qian L, Deguet A, Kazanzides P. ARssist: Augmented reality on a head-mounted display for the first assistant in robotic surgery. Healthc Technol Lett. 2018;5(5):194–200. 10.1049/htl.2018.5065. PMC6372092 30800322

[44] Liu WP, Richmon JD, Sorger JM, Azizian M, Taylor RH. Augmented reality and cone beam CT guidance for transoral robotic surgery. J Robot Surg. 2015;9(3):223–33. 10.1007/s11701-015-0520-5. PMC4634572 26531203

[45] Lin L, Shi Y, Tan A, Bogari M, Zhu M, Xin Y, et al. Mandibular angle split osteotomy based on a novel augmented reality navigation using specialized robot-assisted arms–A feasibility study. J Craniomaxillofac Surg. 2016;44(2):215–23. 10.1016/j.jcms.2015.10.024. 26718052

[46] Wang J, Suenaga H, Yang L, Kobayashi E, Sakuma I. Video see-through augmented reality for oral and maxillofacial surgery. Int J Med Robot. 2017;13(2). 10.1002/rcs.1754. 27283505

[47] Zhou C, Zhu M, Shi Y, Lin L, Chai G, Zhang Y, et al. Robot-assisted surgery for mandibular angle split osteotomy using augmented reality: Preliminary results on clinical animal experiment. Aesthetic Plast Surg. 2017;41(5):1228–36. 10.1007/s00266-017-0900-5. 28725963

[48] Porpiglia F, Checcucci E, Amparore D, Autorino R, Piana A, Bellin A, et al. Augmented-reality robot-assisted radical prostatectomy using hyper-accuracy three-dimensional reconstruction (HA3D™) technology: A radiological and pathological study. BJU Int. 2019;123(5):834–45. 10.1111/bju.14549. 30246936

[49] Madhavan K, Kolcun JPG, Chieng LO, Wang MY. Augmented-reality integrated robotics in neurosurgery: Are we there yet? Neurosurg Focus. 2017;42(5):E3. 10.3171/2017.2.FOCUS177. 28463612

[50] Brockmeyer P, Wiechens B, Schliephake H. The role of augmented reality in the advancement of minimally invasive surgery procedures: A scoping review. Bioengineering (Basel). 2023;10(4):501. 10.3390/bioengineering10040501. PMC10136262 37106688

[51] Bilgic E, Gorgy A, Yang A, Cwintal M, Ranjbar H, Kahla K, et al. Exploring the roles of artificial intelligence in surgical education: A scoping review. Am J Surg. 2022;224(1 Pt A):205–16. 10.1016/j.amjsurg.2021.11.023. 34865736

[52] Vera AM, Russo M, Mohsin A, Tsuda S. Augmented reality telementoring (ART) platform: a randomized controlled trial to assess the efficacy of a new surgical education technology. Surg Endosc. 2014;28(12):3467–72. 10.1007/s00464-014-3625-4. 24962856

[53] Kowalewski KF, Garrow CR, Proctor T, Preukschas AA, Friedrich M, Müller PC, et al. LapTrain: Multi-modality training curriculum for laparoscopic cholecystectomy-results of a randomized controlled trial. Surg Endosc. 2018;32(9):3830–8. 10.1007/s00464-018-6110-7. 29435758

[54] Lee JH, Tanaka E, Woo Y, Ali G, Son T, Kim HI, et al. Advanced real-time multi-display educational system (ARMES): An innovative real-time audiovisual mentoring tool for complex robotic surgery. J Surg Oncol. 2017;116(7):894–7. 10.1002/jso.24722. 28628714

[55] Chowriappa A, Raza SJ, Fazili A, Field E, Malito C, Samarasekera D, et al. Augmented-reality-based skills training for robot-assisted urethrovesical anastomosis: A multi-institutional randomised controlled trial. BJU Int. 2015;115(2):336–45. 10.1111/bju.12704. 24612471

[56] Bertolo R, Hung A, Porpiglia F, Bove P, Schleicher M, Dasgupta P. Systematic review of augmented reality in urological interventions: the evidences of an impact on surgical outcomes are yet to come. World J Urol. 2020;38(9):2167–76. 10.1007/s00345-019-02711-z. 30826888

[57] van Oosterom MN, van der Poel HG, Navab N, van de Velde CJH, van Leeuwen FWB. Computer-assisted surgery: virtual- and augmented-reality displays for navigation during urological interventions. Curr Opin Urol. 2018;28(2):205–13. 10.1097/MOU.0000000000000478. 29278582

[58] Seetohul J, Shafiee M, Sirlantzis K. Augmented Reality (AR) for surgical robotic and autonomous systems: State of the art, challenges, and solutions. Sensors (Basel). 2023;23(13):6202. 10.3390/s23136202. PMC10347167 37448050

[59] Kenngott HG, Wagner M, Gondan M, Nickel F, Nolden M, Fetzer A, et al. Real-time image guidance in laparoscopic liver surgery: First clinical experience with a guidance system based on intraoperative CT imaging. Surg Endosc. 2014;28(3):933–40. 10.1007/s00464-013-3249-0. 24178862

[60] Luo H, Yin D, Zhang S, Xiao D, He B, Meng F, et al. Augmented reality navigation for liver resection with a stereoscopic laparoscope. Comput Methods Prog Biomed. 2020;187:105099. 10.1016/j.cmpb.2019.105099. 31601442

[61] Diana M, Soler L, Agnus V, D’Urso A, Vix M, Dallemagne B, et al. Prospective evaluation of precision multimodal gallbladder surgery navigation: Virtual reality, near-infrared fluorescence, and X-ray-based intraoperative cholangiography. Ann Surg. 2017;266(5):890–7. 10.1097/SLA.0000000000002400. 28742709

[62] Meola A, Cutolo F, Carbone M, Cagnazzo F, Ferrari M, Ferrari V. Augmented reality in neurosurgery: A systematic review. Neurosurg Rev. 2017;40(4):537–48. 10.1007/s10143-016-0732-9. PMC6155988 27154018

[63] Jud L, Fotouhi J, Andronic O, Aichmair A, Osgood G, Navab N, et al. Applicability of augmented reality in orthopedic surgery – A systematic review. BMC Musculoskelet Disord. 2020;21(1):103. 10.1186/s12891-020-3110-2. PMC7023780 32061248

[64] Ayoub A, Pulijala Y. The application of virtual reality and augmented reality in oral & maxillofacial surgery. BMC Oral Health. 2019;19(1):238. 10.1186/s12903-019-0937-8. PMC6839223 31703708

[65] Ciria R, Cherqui D, Geller DA, Briceno J, Wakabayashi G. Comparative short-term benefits of laparoscopic liver resection: 9000 cases and climbing. Ann Surg. 2016;263(4):761–77. 10.1097/SLA.0000000000001413. 26700223

[66] Wakabayashi G, Cherqui D, Geller DA, Buell JF, Kaneko H, Han HS, et al. Recommendations for laparoscopic liver resection: A report from the second international consensus conference held in Morioka. Ann Surg. 2015;261(4):619–29. 10.1097/SLA.0000000000001184. 25742461

[67] Pessaux P, Diana M, Soler L, Piardi T, Mutter D, Marescaux J. Towards cybernetic surgery: Robotic and augmented reality-assisted liver segmentectomy. Langenbecks Arch Surg. 2015;400(3):381–5. 10.1007/s00423-014-1256-9. 25392120

[68] Quero G, Lapergola A, Soler L, Shahbaz M, Hostettler A, Collins T, et al. Virtual and augmented reality in oncologic liver surgery. Surg Oncol Clin N Am. 2019;28(1):31–44. 10.1016/j.soc.2018.08.002. 30414680

